# Fasten: a toolkit for streaming operations on fastq files

**DOI:** 10.21105/joss.06030

**Published:** 2024

**Authors:** Lee S. Katz, John Phan, Henk C. den Bakker

**Affiliations:** 1 Enteric Diseases Laboratory Branch (EDLB), Centers for Disease Control and Prevention, Atlanta, GA, United States of America; 2 Center for Food Safety, University of Georgia, Griffin, GA, United States of America; 3 General Dynamics Information Technology Inc., Atlanta, GA, United States of America

## Statement of need

There are still many gaps in basic command line tools for the handling of standard file formats in the field of bioinformatics. Bioinformaticians have been able to use many tools to manipulate sequence data files in the fastq format, such as seqkit (Shen et al., 2016), seqtk (Li, 2023), FASTX-Toolkit (Gordon, 2014), seqfu (Telatin et al., 2021), jellyfish (Marcais & Kingsford, 2012), or BBTools (Bushnell, 2014). These tools only accept paired-end (PE) sequence data when split into multiple files per sample. Additionally, these tools do not always allow for Unix-style pipe file control. Sometimes they require explicitly input/output options instead of using stdin and stdout. However, some bioinformaticians prefer to combine PE data from a single sample into one file using the interleaved fastq file format, but this format is not always well supported in mainstream tools. Here, we provide Fasten to the community to address these needs.

## Implementation

We leveraged the Cargo packaging system in Rust to create a basic framework for interleaved fastq file manipulation. Each executable reads from stdin and prints reads to stdout and only performs one function at a time. The core executables perform these fundamental functions:1) converting to and from interleaved format, 2) converting to and from other sequence file formats, and 3) ‘straightening’ fastq files to a more standard 4-line-per-entry format.

There are 20 executables including but not limited to read metric generation, read cleaning, kmer counting, read validation, and regular expressions for interleaved fastq files.

## Documentation, testing, and benchmarking

**Figure 1:**
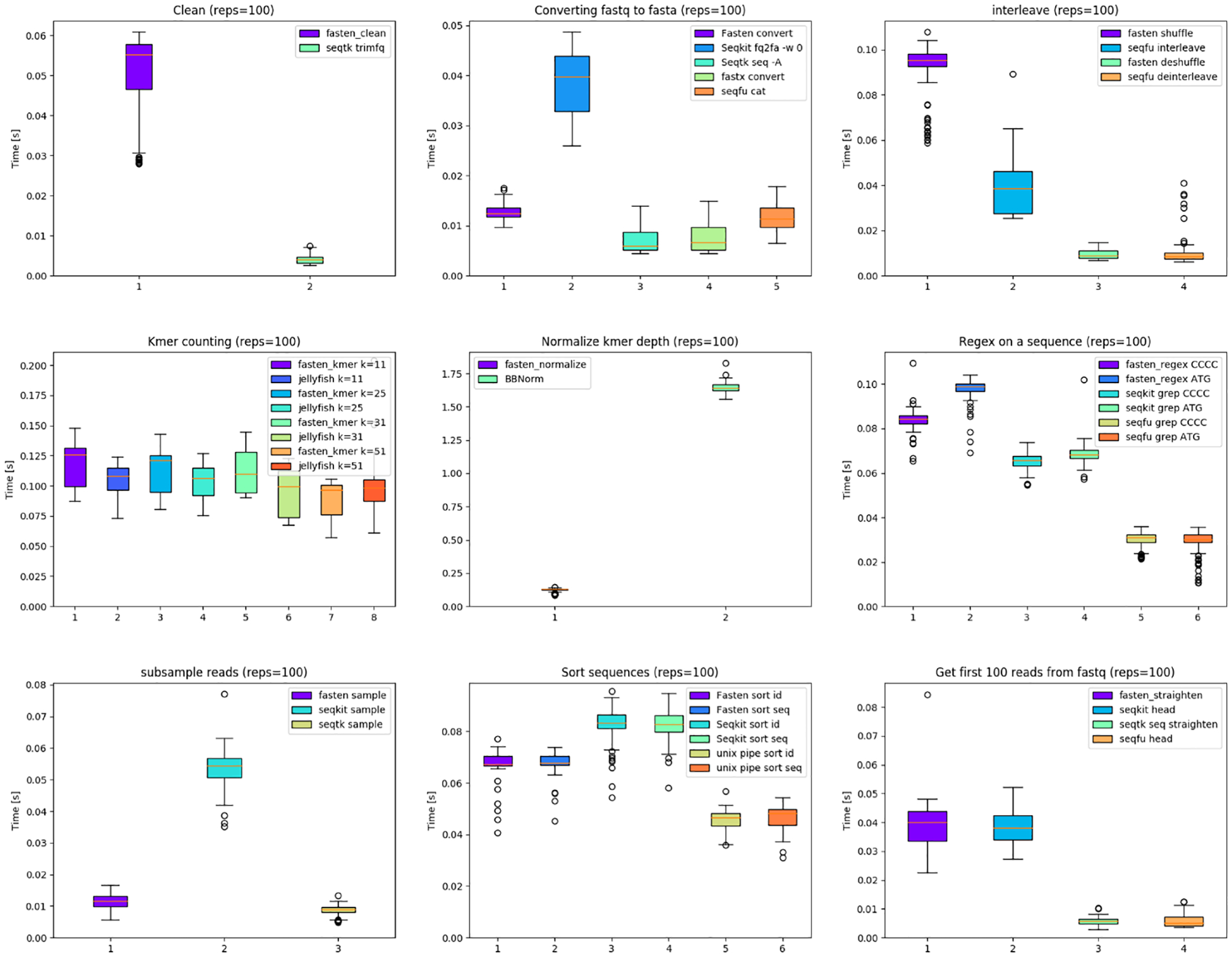
Benchmarks comparing fasten with other analogous tools. From left to right, then to bottom: Trimming with a minimum quality score; converting fastq to fasta; interleaving R1 and R2 reads; kmer counting; normalizing read depth using kmer coverage; searching for a sequence in a fastq file; downsampling reads; sorting fastq entries by either sequence or ID; and converting nonstandard fastq files to a format whose entries are four lines each, and selecting the first 100.

Benchmarking scripts, documentation, the container, and code are available at GitHub. Benchmarking results were graphed into Figure 1. Analogous subcommands from widely used tools were timed and compared against Fasten using the Hyperfine framework (Peter, 2023). Hyperfine options were kept as default, except using 100 replicates and 2 burn-ins. We added inline documentation in the Rust code, which helped make comprehensive and standardized documentation. Continuous integration was implemented in GitHub Actions for unit testing. Each executable is tested to make sure the expected output is obtained with each git push event. We also used GitHub Actions to automatically create a Docker container which is also available on the GitHub repo.

## Conclusions

Fasten is a powerful manipulation suite for interleaved fastq files, written in Rust. We benchmarked Fasten on several categories. It exhibits strengths as shown in Figure 1 but it does not consistently hold the fastest position in all cases. Its major strengths include its competitive speeds, Unix-style pipes, paired-end handling, and the advantages afforded by the Rust language including documentation and stability.

Fasten touts a comprehensive manual, continuous integration, and integration into the command line with unix pipes. It is well poised to be a crucial module for daily work on the command line.
